# Auditory Spatial Recalibration in Congenital Blind Individuals

**DOI:** 10.3389/fnins.2017.00076

**Published:** 2017-02-15

**Authors:** Sara Finocchietti, Giulia Cappagli, Monica Gori

**Affiliations:** Unit for Visually Impaired People, Center for Human Technologies, Fondazione Istituto Italiano di TecnologiaGenoa, Italy

**Keywords:** blindness, auditory localization, visual deprivation, cross-sensory calibration

## Abstract

Blind individuals show impairments for auditory spatial skills that require complex spatial representation of the environment. We suggest that this is partially due to the egocentric frame of reference used by blind individuals. Here we investigate the possibility of reducing the mentioned auditory spatial impairments with an audio-motor training. Our hypothesis is that the association between a motor command and the corresponding movement's sensory feedback can provide an allocentric frame of reference and consequently help blind individuals in understanding complex spatial relationships. Subjects were required to localize the end point of a moving sound before and after either 2-min of audio-motor training or a complete rest. During the training, subjects were asked to move their hand, and consequently the sound source, to freely explore the space around the setup and the body. Both congenital blind (*N* = 20) and blindfolded healthy controls (*N* = 28) participated in the study. Results suggest that the audio-motor training was effective in improving space perception of blind individuals. The improvement was not observed in those subjects that did not perform the training. This study demonstrates that it is possible to recalibrate the auditory spatial representation in congenital blind individuals with a short audio-motor training and provides new insights for rehabilitation protocols in blind people.

## Introduction

The early loss of one sensory input influences the development of the other sensory modalities, e.g., loss of vision impairs audition in the case of blindness (Gori, 2015). As a consequence, the representation of auditory space is partly compromised in congenital blind individuals, suggesting the existence of a trade-off in their auditory localization abilities (King, 2015; Voss et al., 2015). Indeed, some congenital blind individuals show superior performance than blindfolded sighted individuals in discriminating auditory pitch (Gougoux et al., 2004) and relative distance (Ashmead et al., 1998; Voss et al., 2004; Kolarik et al., 2013a,b), creating spatial topographical maps (Tinti et al., 2006; Fortin et al., 2008), and mapping the auditory space in both the peri-personal and extra-personal environment (Lessard et al., 1998; Röder et al., 1999; Voss et al., 2004). This superior performance is especially visible in the case of sound localization in the horizontal plane. On the other hand, congenital blind individuals show worse performance than sighted individuals in localizing sound targets in the vertical mid-sagittal plane (Zwiers et al., 2001; Lewald, 2002), and in a modified version of the mental clock task (Bonino et al., 2015).

In recent studies our group showed that congenital blind individuals are compromised also in performing specific auditory and haptic spatial tasks. In the haptic domain, orientation discrimination (Gori et al., 2010), and arm movement reproduction (Cappagli et al., 2015) result impaired; in the auditory domain, audio depth discrimination in the extra-personal space (Cappagli et al., 2015) and audio space bisection (Gori et al., 2014a) result impaired. The case of space bisection highlights the inexact encoding of Euclidean auditory relationships in blind individuals, since it shows that thresholds for spatially bisecting three consecutive and spatially-distributed sound sources are totally compromised (Vercillo et al., 2015, 2016). Finally, dynamic sound localization - a task which requires a continuous encoding in time and space of a moving sound source - has also been shown to be compromised in the congenitally blind (Finocchietti et al., 2015).

A possible explanation is that in absence of vision, the audio-spatial information can be self-calibrated by the auditory system (Finocchietti et al., 2015). This self-calibration could be sufficient to overcome spatial issues required by some auditory spatial tasks, with changes within the auditory pathway and the recruitment of the visual cortex (Collignon et al., 2009; Merabet and Pascual-Leone, 2009), but it is probably insufficient to compensate for others spatial aspects as the ones that require the development of metric representations (as suggested by Gori et al., 2014a).

It has been recently shown that audio spatial perception can be improved in sighted individuals by audio-tactile associations (Gori et al., 2014b) and by visual exploration (Tonelli et al., 2015). In addition, echolocation, i.e., the ability to produce self-generated sounds to measure the time delay between their own sound emission and any echo reflected by the environment, helps to improve the spatial perception in blind individuals (Vercillo et al., 2015). Since recent findings suggest that sighted children acquire spatial capabilities thanks to the reciprocal influence between visual perception and execution of movements (Bremner, 2008), an interesting question is whether audio-motor feedback can be used to recalibrate auditory spatial maps in blind individuals. Here we investigated if an audio-motor training can improve complex aspects of auditory space perception in blind and sighted individuals. We tested the performance of congenital blind individuals and sighted controls in a dynamic auditory localization task before and after an audio-motor training. Our results support the aforementioned hypothesis showing that even a short audio-motor training can improve audio space perception in blind individuals.

## Methods

### Subjects

Forty-eight participants have been enrolled in the study: Congenital blind (*N* = 20; 13 females, mean age 42 ± 12 years old), and sighted blindfolded controls (*N* = 28; 14 females, mean age 40 ± 16 years old). All the participants had similar education (at least an Italian high school diploma, indicating 13 years of school). The blind participants were congenital blind and the vision loss had different etiology (Table 1).

**Table 1:** **Clinical details of the congenital blind (CB) participants**.

**Subject**	**Gender**	**Age**	**Pathology**	**Age complete blindness**	**Residual vision at test**
**WITHOUT TRAINING**					
CB1	F	27	Retinitis pigmentosa	At birth	Lights and shadows
CB2	F	32	Congenital cataract	Before birth	No vision
CB3	F	32	Retinopathy of Prematurity	Before birth	No vision
CB4	F	39	Congenital cataract	Before birth	Lights and shadows
CB5	F	53	Eyeball atrophy	Before birth	No vision
CB6	F	54	Retinitis pigmentosa	Before birth	Lights and shadows
CB7	M	43	Leber's amaurosis	Before birth	No vision
CB8	M	56	Uveitis	Before birth	Lights and shadows
CB9	M	57	Retinopathy of Prematurity	Before birth	No vision
CB10	M	57	Congenital glaucoma	Before birth	No vision
**WITH TRAINING**					
CB1	F	20	Congenital glaucoma	Before birth	No vision
CB2	F	25	Leber's amaurosis	Before birth	No vision
CB3	F	28	Retinopathy of Prematurity	Before birth	No vision
CB4	F	37	Retinopathy of Prematurity	Before birth	No vision
CB5	F	39	Congenital cataracts	Before birth	Lights and shadows
CB6	F	42	Congenital cataract	Before birth	Lights and shadows
CB7	F	57	Atrophy of the eyeball	Before birth	No vision
CB8	M	48	Retinopathy of Prematurity	Before birth	No vision
CB9	M	49	Fibroplasya	Before birth	No vision
CB10	M	50	Retinopathy of Prematurity	Before birth	No vision

*The table shows the age at test, the gender, the pathology, and eventual residual vision*.

All the participants had normal hearing (assessed by audiometric test), no cognitive impairments, and were right handed (Oldfield, 1971). The participants provided written informed consent in accordance with the Declaration of Helsinki. The study was approved by the ethics committee of the local health service (Comitato Etico, ASL3 Genovese, Italy).

### Set-up and task

**Figure 1:**
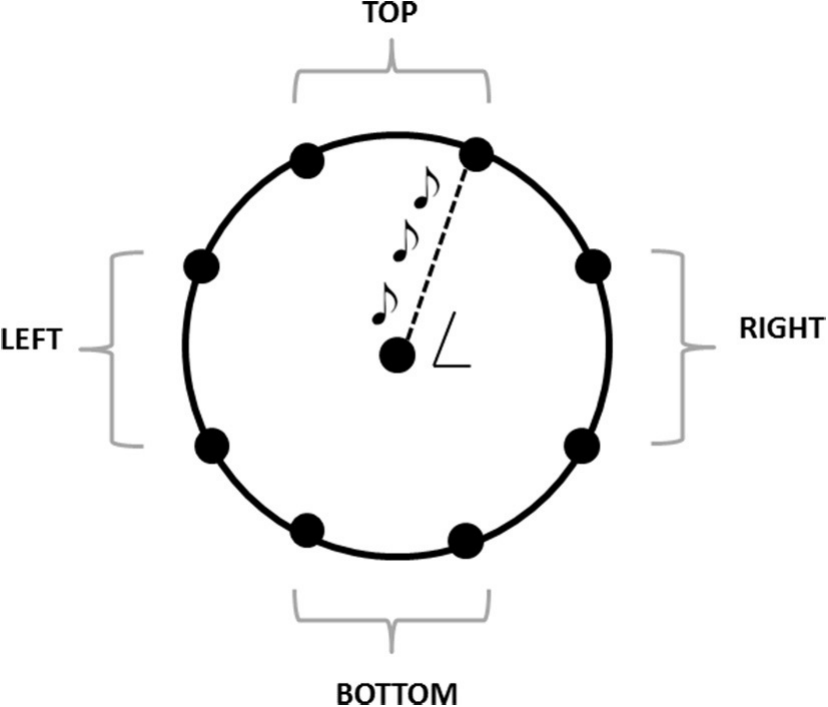
**The graduated circular perimeter (radius = 45 cm) is mounted on a wooden panel positioned in front of the participant on the frontal plane**. The eight different positions are marked on the perimeter starting at 22.5° and increasing of 45°. The central position corresponded at the tip of the nose of the participant. For the analysis, the eight points are grouped in 4 areas: Top, left, bottom, right.

The experiment was performed in a dark room. The apparatus consisted of a graduated circular perimeter (radius = 45 cm) mounted on a wooden panel positioned in front of the participant on the frontal plane. Eight different positions were marked on the perimeter, starting at 22.5° on the y-axis and separated by increments of 45° (Figure 1). Sighted participants were blindfolded before entering the experimental room. Each participant was seated, the center of the circle corresponding to the tip of his nose, and was able to comfortably reach and explore with his hand the graduated circular perimeter. One of the two experimenters instructed the participant and performed all the experiments (SF, GC). Both experimenters were previously trained to administer the task as similar as possible. The experimenter was seated opposite to the participant, holding the sound source with the speaker toward the subject, making the sound clearly audible by every participant. The sound was a single pulse at 500 Hz, intermittent sound at 180 bpm, as previously used by our group (Finocchietti et al., 2015). A spherical marker was mounted on the distal phalanges of the index finger on both the participant and the experimenter for motion tracking (Vicon Motion Systems Ltd., UK). The experimenter moved the sound source from the center of the plane toward one of the possible positions highlighted on the circular perimeter in a randomized order. Moving sounds were presented in 2D in the vertical plane (see Figure 1). The participant was instructed to keep his index finger pointed to the center until the end of the audio motion and to keep the head as still as possible during all the session. He then had to reproduce the complete trajectory, reach the estimated sound end-point position, and return to the original central position. The movement was performed at participant's own pace. All the eight positions were reached five times, for a total of 40 trials per participant.

### Protocol

The dynamic auditory localization task previously described was performed in two sessions. After the first session, subjects were randomly assigned to one of two groups using a Matlab (R2013a, The MathWorks, USA) built-in function. The first group performed the audio-motor training with the sound source for 2 min. The short timing for the audio-motor training was chosen to show that the recalibration can happen really fast, as soon as the presence of an auditory feedback associated to a movement allows to restore an allocentric frame of reference. The second group rested for the same duration without any other instruction. During the training, subjects of the first group were holding the sound source and were asked to move their hand, and consequently the sound, to freely explore the space around the setup and the body. The setup was removed during this exploration. Afterwards both groups performed the second session of the hand pointing task.

### Data analysis and statistics

Kinematic data were post-processed and analyzed using Matlab (R2013a, The MathWorks, USA). Each end-point position was computed as the average of the last 10 samples and normalized on the origin position (the center of the circumference), in order to avoid movement errors. Spatial accuracy, measured as localization error, and precision, calculated as standard deviation of the measurements, were obtained for each participant and each spatial position. The localization error was calculated as the Euclidean distance (in mm) between the end-point position reached by the participant and the position reached by the experimenter. This error was averaged on the number of trials per position and on the number of participants. The accuracy was calculated as standard deviation for each point and averaged among subjects. Data were normally distributed, confirmed by visual inspection of Q–Q plots. Data are presented as mean and standard error (SE). The eight points were grouped in 4 panel areas, as indicated in Figure 1. The accuracy, precision, mean velocity and path length were analyzed with a factorial ANOVA, with between-factors training group (two levels: Training. no training), participant group (two levels: CB, controls), and within-factors session (two levels: Pre, post), panel area (four levels: Top, left, right, bottom). The Bonferroni *Post-hoc* test was used in the case of significant factors (*p* < 0.05 was considered significant).

## Results

### Accuracy

**Figure 2:**
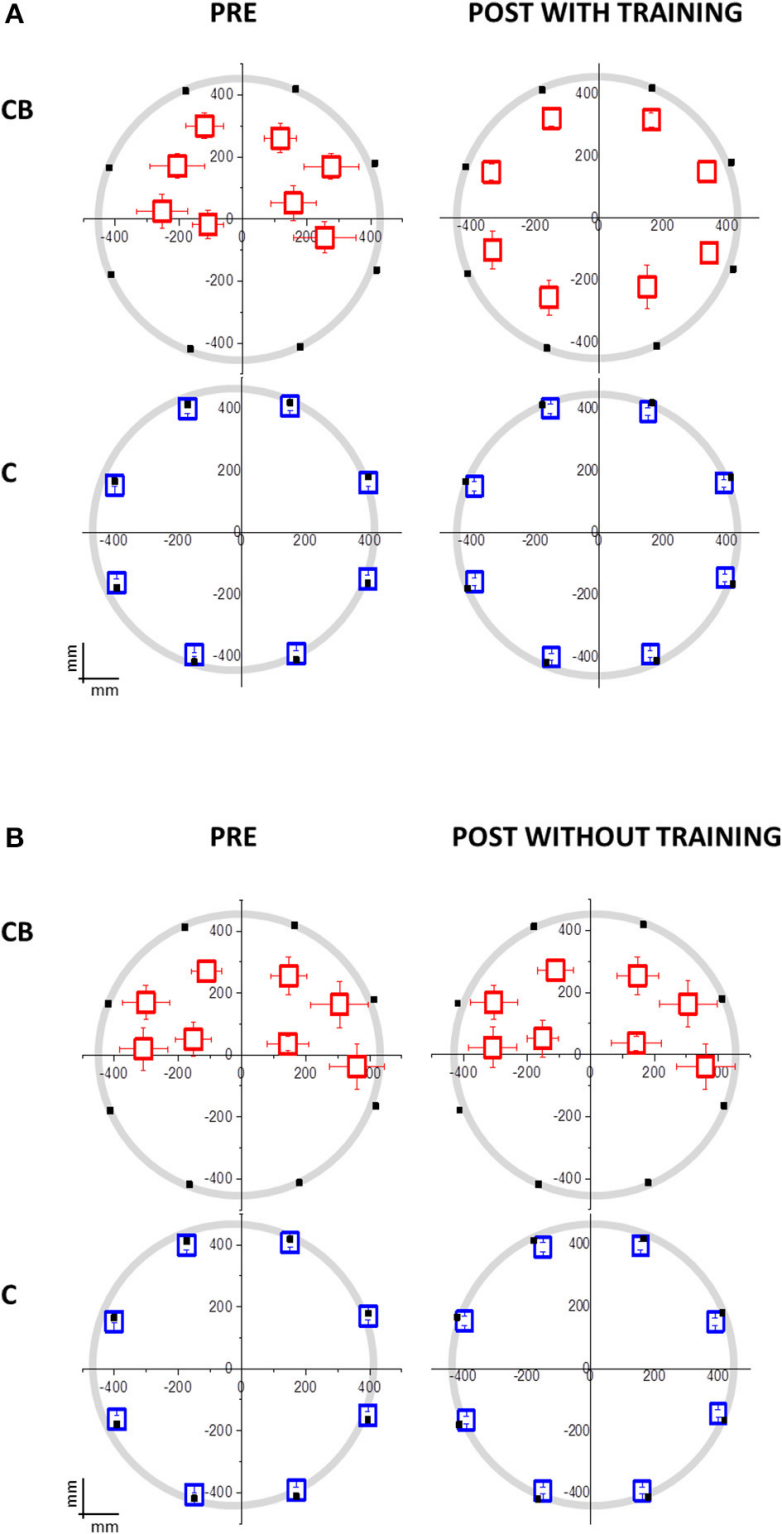
**Mean localization bias in congenital blind individuals (CB) and sighted blindfolded controls (C) relative to the hand pointing task following the moving sound from the origin to one of the eight position on the circle**. The black dots indicate the 8 possible end-point positions. The origin (0,0) corresponds to the nose of the participant. **(A)** Group that performed the audio-motor training (CB: *N* = 10; 7 females; mean age = 39 ± 12 years old; C: *N* = 14; 7 females; mean age = 35 ± 15 years old). The deficit in the lower side positions isn't present in CB after the training. No significant change is present in C. **(B)** Group that did not perform the audio-motor training (CB: *N* = 10; 6 females; mean age = 45 ± 12 years old; C: *N* = 14; 7 females; mean age = 44 ± 13 years old). The deficit in the lower side positions is maintained in CB after the inter-session without training. No difference is present in C, between pre and post-session, and between training group.

Regarding the first session, congenital blind individuals showed worse spatial accuracy than sighted blindfolded controls (interaction between panel area x participant group x session, [*F*_(3, 32)_ = 7.91, *p* = 0.0004, Figure 2]. The deficit was present for all the spatial positions considered, even if it was more pronounced for the lower positions of the plane (Bon: *p* < 0.001).

**Figure 3:**
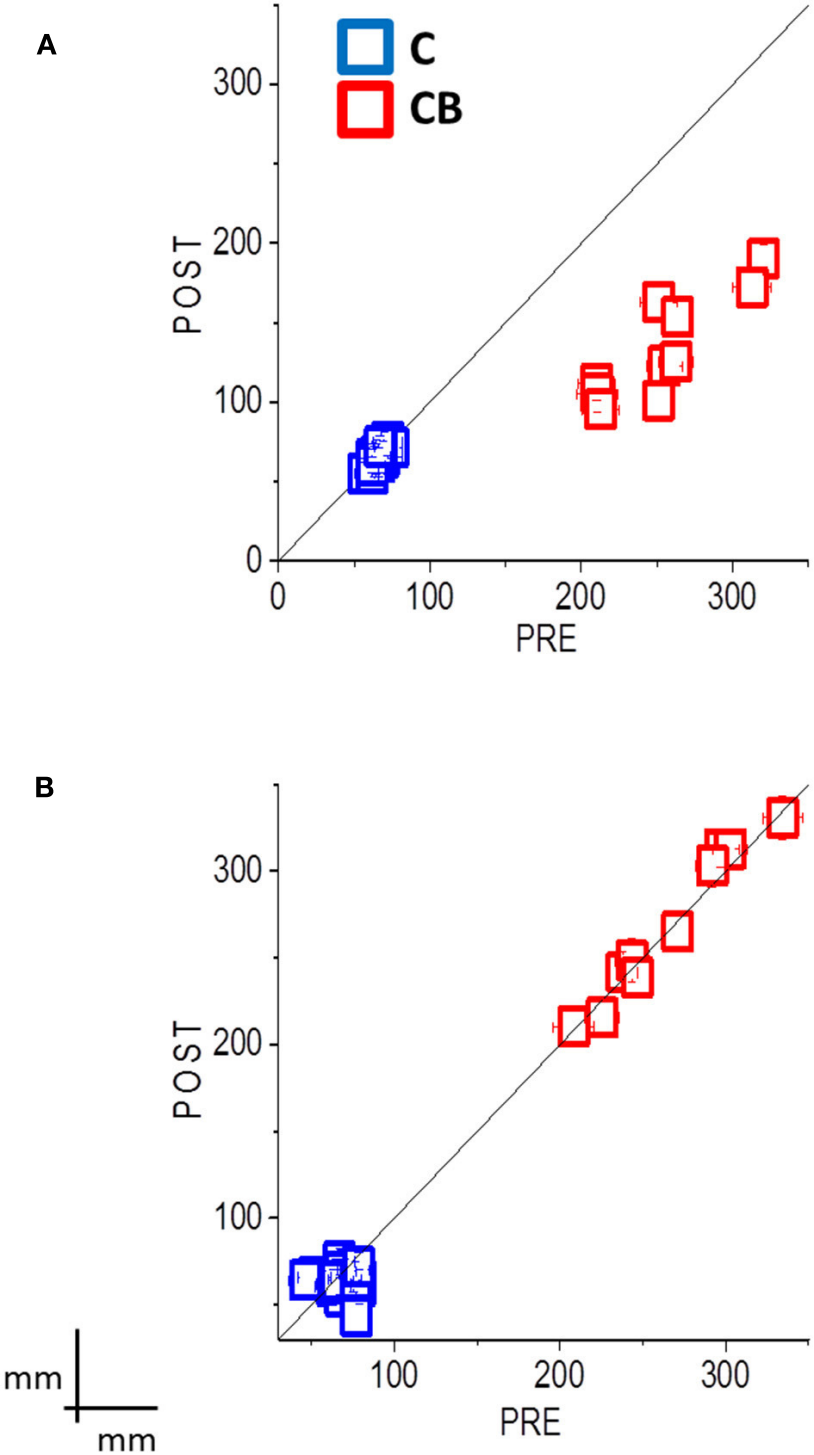
**Summary data shows the mean localization error (±SE) for all subjects, pre-session results plotted against post-session results, with audio-motor training (A)** and without audio-motor training **(B)**. Sighted blindfolded controls (C) are in blue, while congenital blind subjects (CB) are in red. If the localization error is decreased after the inter-training session, the data should fall below the black equality line. This happens to the CBs after the audio-motor training, where the localization error reduces between 49 and 55%. The data instead fall along the black equality line for the group without the audio-motor training, showing a mean localization error between 21 and 33 cm. Controls always showed an error less than 10 cm, with no improvement in the performance.

Regarding the second session, the group of congenital blind subjects that performed the training improved in spatial accuracy [interaction between panel x participant group x session x training *F*_(3, 32)_ = 7.65; *p* = 0.001; Bon: *p* < 0.01, Figure 3A, red dots]. In fact, the performance of the congenital blind group became similar to the one of sighted individuals, even if the spatial distribution of the pointing responses was not as accurate as the one of the sighted blindfolded group (Figure 2A, right panels). The spatial accuracy of the congenital blind group that did not perform the audio-motor training did not improve (Bon: *p* > 0.1, Figure 2B right panels). Sighted blindfolded controls did not further improve their performance (Bon: *p* > 0.2), as their data remained above the equality line in Figure 3.

### Precision

The precision was comparable among factors for both early blind and sighted blindfolded controls, independently of the audio-motor training [interaction between panel area x participant group x session x training *F*_(1, 32)_ = 4.74; *p* > 0.3]. In fact, the precision was 34 ± 14 mm for the congenital blind groups, and 30 ± 15 mm for the sighted blindfolded groups.

### Velocity

Every participant was free to perform the movement at his own pace, but no difference in mean velocity among conditions was observed [interaction between panel area x participant group x session x training: *F*_(3, 32)_ = 3.56, *p* > 0.1, Table 2].

**Table 2:** **Average velocity [m/s] between blind and controls for the different conditions**.

	**With training**		**Without training**	
	**Pre**	**Post**	**Pre**	**Post**
**BLIND**				
Top	0.934 ± 0.027	1.107 ± 0.020	1.316 ± 0.022	1.244 ± 0.019
Left	1.223 ± 0.025	1.035 ± 0.021	1.232 ± 0.019	1.048 ± 0.021
Right	0.913 ± 0.021	0.986 ± 0.025	1.325 ± 0.023	1.325 ± 0.20
Bottom	1.312 ± 0.019	1.002 ± 0.019	0.917 ± 0.019	0.831 ± 0.29
**CONTROL**				
Top	1.310 ± 0.022	0.977 ± 0.020	1.114 ± 0.021	1.114 ± 0.025
Left	0.937 ± 0.024	1.313 ± 0.019	0.889 ± 0.024	1.010 ± 0.029
Right	1.000 ± 0.021	1.251 ± 0.021	0.958 ± 0.019	1.074 ± 0.019
Bottom	1.174 ± 0.025	0.898 ± 0.025	0.877 ± 0.022	1.294 ± 0.021

*No statistical difference was present, as the range of velocity was between 0.0889 and 1.325 m/s with standard deviation between 0.018 and 0.029 m/s*.

### Path length

For the blind group the path length resulted longer in the post-training session, in comparison to the first session and to the group that did not perform any training [*F*_(3, 32)_ = 5.49; *P* = 0.03; Bon: *P* = 0.05, Table 3]. In this last group, the bottom area always showed shorter path length than the other three areas, independently of session and group training, showing an average path length of 0.25 m. Control individuals showed a longer path length in comparison to the blind individuals, independently of session, group training, and panel area [*F*_(3, 32)_ = 7.38; *P* = 0.05; Bon: *P* = 0.04, Table 3].

**Table 3:** **Average path length [m] between blind and controls for the different conditions**.

	**With training**		**Without training**	
	**Pre**	**Post**	**Pre**	**Post**
**BLIND**				
Top	0.265 ± 0.025	0.381 ± 0.025^*^	0.270 ± 0.022	0.274 ± 0.020
Left	0.273 ± 0.021	0.389 ± 0.011^*^	0.275 ± 0.020	0.278 ± 0.021
Right	0.268 ± 0.020	0.394 ± 0.017^*^	0.281 ± 0.023	0.281 ± 0.23
Bottom	0.219 ± 0.019#	0.351 ± 0.019^*^#	0.224 ± 0.019#	0.225 ± 0.24#
**CONTROL**				
Top	0.427 ± 0.022	0.432 ± 0.016	0.434 ± 0.018	0.428 ± 0.016
Left	0.435 ± 0.017	0.440 ± 0.010	0.438 ± 0.022	0.429 ± 0.020
Right	0.441 ± 0.011	0.428 ± 0.021	0.439 ± 0.019	0.438 ± 0.019
Bottom	0.428 ± 0.025	0.431 ± 0.017	0.440 ± 0.020	0.436 ± 0.021

Blind individuals: the path length was longer in the blind group that performed the audio-motor training in comparison to the first session and the group that did not perform any training (indicated with ^*^). The bottom area always showed shorter path length than the other three areas, independently to session and group training (indicated with #). Control individuals: a longer path length in comparison to the blind participants was always presented, and independently to session, group training, and panel area.

## Discussion

The brain continuously integrates sensory and motor inputs to optimize environmental perception and interaction (Ernst and Banks, 2002). For example, when performing an action sighted individuals merge the visual and the proprioceptive inputs to create a mental representation of their body movements. When vision is absent, the sensory feedback of body movement is not generally provided and this form of sensory-motor integration is not possible.

We recently showed, in agreement with other authors (Zwiers et al., 2001; Lewald, 2002; Voss et al., 2015), that blind individuals have problems in perceiving specific spatial relationship between sounds or moving sounds in a 2D space (Gori et al., 2014a; Cappagli et al., 2015; Finocchietti et al., 2015). This is possibly associated with the evidence that congenitally blind individuals seem to process the spectral cues for sound localization differently than sighted individuals (Lewald, 2002; Voss et al., 2015).

Here we studied if an audio-motor feedback can improve audio spatial perception by reducing the spatial deficits observed in blind individuals. We asked blind individuals to perform a dynamic auditory localization task before and after a short (2 min) audio-motor training, in which they had to freely move their hand with a sound source positioned on the wrist. We showed a strong improvement in the ability to localize a moving sound source in blind individuals after the training. In particular, an improvement of 49–55% in the accuracy has been found for blind individuals (see in Figure 3, all the data fall below the equality line). The results on average velocity and path length confirm this result: Blind individuals showed a similar average movement velocity than controls, but fail in correctly encoding the distance between the starting point and the end point of a moving source without a proper audio-motor training. This improvement mainly affects the vertical direction, which is the dimension typically impaired in blind individuals (Zwiers et al., 2001; Lewald, 2002; Voss et al., 2015).

These results show that the audio-motor training can recalibrate the auditory space perception in blind individuals, possibly because it allows to collect some allocentric information coming from the audio-motor association provided by the training.

Indeed the human brain makes use of egocentric or allocentric coordinates to obtain different perspectives of the environment (Avraamides et al., 2004). For example, the position of an object in the space can be represented respect to oneself (egocentric frame of reference, FoR) or in object-centered coordinates that are independent of the observer's current position (allocentric FoR). Previous studies have shown an inhibition of the FoR in blind individuals (Röder et al., 2007; Pasqualotto and Proulx, 2012). When a sighted person moves his hand, he can perceive his own movement thanks to the combination of proprioceptive and visual inputs that can mediate the mapping between body centered and space centered coordinates. With the lack of vision, the visual feedback of actions is missing. On the other hand, for blind individuals the sensory feedback of body movements can be provided by the auditory system thanks to an audio source positioned in the hand. In this way the auditory feedback of body movements can be used to map spatial and body coordinates. A possible explanation of our results is that the audio-motor training might have caused a shift from an egocentric to an allocentric reference frame stimulated by the auditory feedback of body movements, thus improving the overall perception of auditory signals in the space. In agreement with this idea it has been repeatedly suggested that the emergence of allocentric coding coincides with the beginning of self-locomotion in typically developing infants (Clearfield, 2004; Ricken et al., 2004). The subsequent increase in spatial abilities strongly relies on further experience with independent locomotion and exploration, which usually provides the opportunity to encode events and stimuli from different perspectives (Lew et al., 2000; Vasilyeva and Lourenco, 2012).

To conclude, we suggest that sensory feedback of body movements are important for the development of spatial skills and that it is possible to improve spatial abilities in congenital blind individuals thanks to non-visual sensory feedback of motor signals. We think that this kind of audio-motor feedback can substitute the visuo-motor feedback and recalibrate specific spatial abilities that might require an allocentric frame of reference.

It is not clear to date which is the neural mechanisms involved in the fast perceptual recalibration mechanism that we observed. Further studies will be necessary to better describe this process and identify the neural mechanism supporting it. The findings of the present study represent an important input for the development of new rehabilitative protocols meant to improve basic and advanced sensory skills in individuals with visual disability.

## Author contributions

SF, GC, and MG: Designed the research; SF and GC: Performed the research; SF: Analyzed the data; SF, GC, and MG: Wrote the article.

### Conflict of interest statement

The authors declare that the research was conducted in the absence of any commercial or financial relationships that could be construed as a potential conflict of interest.
