# Corrigendum: Auditory Spatial Recalibration in Congenital Blind Individuals

**DOI:** 10.3389/fnins.2017.00268

**Published:** 2017-05-09

**Authors:** Sara Finocchietti, Giulia Cappagli, Monica Gori

**Affiliations:** Unit for Visually Impaired People, Center for Human Technologies, Fondazione Istituto Italiano di TecnologiaGenoa, Italy

**Keywords:** blindness, auditory localization, visual deprivation, cross-sensory calibration

There is an error in the Funding statement, the name of the funder is incorrect. The correct funding statement is as follows. **This research has been supported by the European ABBI project (FP7-ICT 611452)**. The authors apologize for this error and state that this does not change the scientific conclusions of the article in any way.

## Conflict of interest statement

The authors declare that the research was conducted in the absence of any commercial or financial relationships that could be construed as a potential conflict of interest.

